# Supplementary material for: Impact of vitamin D status and cathelicidin antimicrobial peptide on adults with active pulmonary TB globally: A systematic review and meta-analysis
Source: PLoS One. 2021 Jun 11;16(6):e0252762. doi: 10.1371/journal.pone.0252762 (PMC8195352; doi:10.1371/journal.pone.0252762)
Supplement: S2 File — (PDF) [file pone.0252762.s002.pdf]

PROTOCOL FOR SYSTEMATIC REVIEW

Review team

Ester Lilian Acen, Irene Andia Biraro, William Worodria, Moses Joloba, Bill Nkeeto, David Kateete.

Affiliations

1 Physiology department school of Biomedical Sciences

Makerere University College of Health Sciences

2Head of Immunology and Molecular Biology Department

Makerere College of health sciences muk

Email: dkateete@med.mak.ac.ug Telephone 0712875725

2 Dean School of Biomedical Sciences

Makerere College of health sciences

Email: mosesjoloba@case.edu telephone 0782752582

3Infectious Diseases Institute, College of Health Sciences, Makerere University, Kampala, Uganda,

Infectious Diseases Division Department of Internal Medicine

Makerere College of Health Sciences

Email: andiaodanga@yahoo.com telephone 070039080

4 Pulmonary, Division, Department of Internal Medicine Mulago National Referral Hospital

Email: Worodria @yahoo.com Telephone 0772424601

Type of systematic review:

This will be a Quantitative-systematic review

Title of review:

The impact of vitamin D status and Cathelicidin antimicrobial peptide on adults with Pulmonary TB disease: A systematic review

Aim of the Review:

The aim of this review is to determine the association of vitamin D status with cathelicidin expression among adults with pulmonary TB disease.

Introduction

Tuberculosis (TB) remains a global threat and a public health problem notwithstanding attempts to eradicate it. One third of the world population is able to sustain equilibrium between the host and *MTB* consequently remaining latent; however these maintain a large pool of individuals for progression to disease. First and foremost a clear understanding of the mechanisms involved in reactivation from latent infection to active TB disease may be a better strategy towards elimination of TB disease. Accordingly a component of recent TB research has focused on host directed therapy, in which understanding the mechanisms of host immune response is utilized to fight the pathogen. Cathelicidin (LL-37) antimicrobial peptide is involved in both innate and adaptive immunity moreover both the innate and adaptive immune pathways play a role in the host immune response. This antimicrobial peptide (AMP) exhibits an important function through direct killing of the pathogen. LL-37 is up regulated in a vitamin D dependant manner and therefore its direct killing mechanism is dependent on vitamin D status. The association of vitamin D with tuberculosis is demonstrated by the increase in LL-37 production and enhanced activity of the macrophages leading to MTB destruction (Ho-Pham et al., 2010) A study by (Rivas-Santiago et al., 2008,) suggested that LL-37 is involved in innate immunity after the discovery that macrophages profoundly expressed LL-37 in early TB infection. Another study among healthy individuals found a correlation of LL-37 and 25(OH)D and consequently an increase in levels after administration with VD3 (Liu et al., 2006). In contrast a study carried out among the elderly reported no correlation between circulating 25(OH)D and LL-37 probably due to confounding factors in this age group (Adams et al., 2009). Furthermore a multicenter study performed among USA and Canadian TB patients reported severe vitamin D deficiency, however did not find a correlation between vitamin D levels and LL-37 (Yamshchikov et al., 2010). Besides another study that examined the expression of LL-37 mRNA by TLR2/1 found low LL-37 levels among black race individual samples as compared to the white individuals' samples. This may explain the frequency and severity of TB disease in the black race compared to the white race (Stead et al., 1990). Moreover the black or African race has genetic

polymorphisms that may predispose them to high utilization of vitamin D leading to low circulatory levels. Generally although vitamin D has been found to have therapeutic properties the underlying mechanisms have not been clearly delineated. LL-37 molecule activated through the vitamin D metabolic pathway could be the closer answer to this puzzle. However have been contrasting findings worldwide on the association of vitamin D levels with LL-37 antimicrobial peptide. This review study attempts to define the relationship between LL-37 levels and vitamin D status in pulmonary TB infection and disease. Therefore this information may help in provide a clear relationship between these two molecules hence bridge the gap in this area of research.

Research questions:

1) Is there a difference in vitamin D levels and LL-37 levels among adults with TB disease and those without disease?

2) Is there a relationship between vitamin D status and LL-37 levels among adults with TB and those without disease?

Population: pulmonary TB individuals

Scope: Globally

Study designs

We shall aim to include case control studies and comparative cross sectional studies and centred on association of LL-37 levels or expression and vitamin status in adult with active pulmonary TB and their controls in any country worldwide.

Inclusion and exclusion criteria

We shall include articles with studies whose participants were enrolled from a hospital setting or clinic in which patients seek medical assistance worldwide. We shall include all studies highlighting information such as association, correlation, relationship, impact or effect of vitamin D status on LL-37 expression in pulmonary TB infection and disease. We will restrict the languages to English and French since it is widely spoken. However related abstracts in other languages with English interpretation will be included. There will be no restriction to year of publication. We shall exclude studies among children, cells and animal models, clinical trails, prospective studies cohort studies, case reports/ series, studies of vitamin D levels and LL-37 in

other patients' populations other than pulmonary TB. However studies reporting on vitamin D and LL-37 levels in a population of pulmonary TB together with another disease will be included. For this review, we shall define hypovitaminosis D vitamin D levels as ≥ 20 ng/ml – 29 ng/ml. Those with ≥ 20 ng/ml will be categorized as vitamin D deficiency and those between 21-29 ng/ml will be vitamin D insufficient and those with ≥30 ng/ml will be categorized as vitamin D sufficient.

Databases:

PubMed, HINARI, Google Scholar and EBSCO host

Key search words:

- Cathelicidin levels
- LL-37
- Antimicrobial peptide
- Hypovitaminosis D
- Vitamin D deficiency
- Serum/plasma Vitamin D levels
- 25(OH)D levels
- Tuberculosis
- Pulmonary
- Mycobacterium infection

Search strategy

We shall perform a PubMed electronic search to identify primary studies addressing LL-37 expression with hypovitaminosis D, vitamin D status, vitamin D deficiency. In the first search, we shall use the term cathelicidin OR LL-37 AND other individual key words; Tuberculosis, Hypovitaminosis D, vitamin D deficiency; severe deficiency, serum/ plasma hydroxyl vitamin D, 25(OH) D. We shall then repeat the search in HINARI and Google scholar, and EBSCO host to obtain more references. We shall then search reference lists from retrieved articles. Any grey data available will be searched by contacting authors after perusal from related conference proceedings between 2010- 2018.

Review of studies

A database will be created from the electronic searches and kept in Endnote X7 programme. Two reviewers will screen the citations using title and abstract without blinding to capture relevant studies. The database will then be screened again using full text for each study to include only primary relevant articles. We shall only include studies addressing LL-37 and vitamin D levels with pulmonary, LL-37 expression and association with vitamin D levels in TB, vitamin D hypovitaminosis or deficiency LL-37in patients with pulmonary TB in any country of the world.

Data summary and analysis

Data from the final studies will be summarised in an excel spreadsheet, recording information such as; title, first author, year of publication, country/city latitude, study type, sample size, population, TB cases; controls, age; laboratory test; predictor variables; percentages of males and females; and serum cuff of levels , means and standard deviations or medians and interquartile , association/correlation of cathelicidin and vitamin D, levels or Relationship of low vitamin D levels and cathelicidin expression. All articles displayed on that search engine will be sent to Endnote. Later the reference Manager will be checked and all articles with irrelevant information to the topic will be excluded. This data will then be transferred to STATA (Stat Corp. STATA15.0, College Station, Texas USA.

Timeframe

The review is expected to take 9 months to complete.

Bias

The grading of Recommendations and Assessment Development and Evaluation (GRADE) approach will be utilized to assess the quality of evidence. During data extraction process two reviewers will independently gather information from primary studies, and will asses for risk of bias by screening raw data to check for completeness of information and any other anomalies that may follow. Disagreements from the two reviewers will be resolved by a third reviewer. Quality of the primary studies to be included in this systematic review will be of utmost importance. Studies with reasonably valid association results will be included. Only studies with comparable methodological, clinical and statistical quality and any other necessary information will be selected.

Synthesis

A narrative synthesis with evaluation of individual primary studies will be done. Chi square test will be used for analysis of heterogeneity using STATA (Stat Corp. STATA12.0, College Station, Texas USA. In case of homogenous data quantitative synthesis will be performed and continuous variables will be analysed as means and standard deviations or medians and interquartile range. Heterogeneity among the primary studies will be assessed using a random effect model. Results will be presented as forest plots. Depending on the variation assessed by this model decision on whether to further perform a Meta analysis will be made.
