# Supplementary material for: Impact of vitamin D status and cathelicidin antimicrobial peptide on adults with active pulmonary TB globally: A systematic review and meta-analysis
Source: PLoS One. 2021 Jun 11;16(6):e0252762. doi: 10.1371/journal.pone.0252762 (PMC8195352; doi:10.1371/journal.pone.0252762)
Supplement: Attachment — Submitted filename: Response to Reviewers.docx [file pone.0252762.s004.docx]

**Response to Reviewers**

Dear Academic Editor

Please find response to my review.

Impact of vitamin D status and cathelicidin antimicrobial peptide on adults with active pulmonary TB globally: A systematic review and meta-analysis

**Additional Editors comments**

1.Please update the last search to allow the inclusion of studies published in the past 12 months.
Please ensure you have included the full electronic search strategy for at least one database and uploaded it as an additional file.

Response: a latest search in PubMed has been attached

Please reformat table 1 in landscape

Define Nc, Please present as NC or Nc, currently both used

Define PTB, PBMC

Response

Line 221 Table one has been reformatted to landscape

Line 82 have defined PTB -

Line 207 PBMC

Line 100 have defined NC

- line 221Presented as NC in table 1

**Reviewer 1**

1. “The objective of our systematic review was to compare vitamin D and LL-37 levels among TB patients to non-pulmonary TB (…) and to determine the association between vitamin D and LL-37 and any ….”

This last aim is clear and reflects the performed study so would suggest adjusting the previous ones

Response

Line 27 The aim was adjusted according to suggestion -

2. There are two different types of studies, one is the determination of LL-37 plasma levels and the other is the cell expression of LL-37. It could be more explicit in the text. Also, for Vitamin D.

Response;

Line -74 Expression of mRNA in cells as been	included

3. The lowest vitamin D levels of >10ng/ml (…) six studies reported vitamin D deficiency levels of >20ng/ml”. This statement is not clear.

Response

Lines 232-234 Sentences have been clearly written

4. Table 1. Would suggest a reference in author column. It will help the readers find the article.

Response

Line 220 Table one has been modified to include author

5. Suggest reviewing the references. Some are not complete (for example reference 8)

Response

Line 438-439 Reference has been completed

Checked bibliography and no retracted articles

**Reviewer 2**

1. Please clarify which is the target of this study – TB or active pulmonary TB

Response

Line 99-100 The population is pulmonary TB patients however pulmonary TB is active TB

2. Please add some data in the abstract’s result section.

Response

Line 37 additional data

Line 40 additional data

3. the introduction is too long

Line 75 sentence removed to reduce introduction

Line 77 sentence removed to reduce introduction

4. Please add the level of Vit D and LL-37 and case number in TB and non-TB in the table 1.

Response

Line 221; table has been modified to include those variables
